# Bacillus Calmette-Guérin immunotherapy-induced extracellular vesicles promote antitumor immunity in bladder cancer

**DOI:** 10.3389/fimmu.2026.1707754

**Published:** 2026-04-13

**Authors:** Carlos J. Ortiz-Bonilla, Ryan D. Molony, Hiroshi Miyamoto, Edward Messing, Yi-Fen Lee

**Affiliations:** 1 Department of Pathology and Laboratory Medicine, University of Rochester Medical Center, Rochester, NY, United States; 2 Department of Urology, University of Rochester Medical Center, Rochester, NY, United States

**Keywords:** BCG immunotherapy, biomarker, bladder cancer, CD8+ T cells, extracellular vesicles

## Abstract

**Background:**

Intravesical Bacillus Calmette-Guérin (BCG) immunotherapy remains one of the most effective treatments for non-muscle invasive bladder cancer (NMIBC). However, a substantial proportion of patients fail to respond, and the precise mechanisms underlying BCG’s therapeutic effects remain incompletely understood. While extracellular vesicles (EVs) can modulate a range of biological processes, their relevance in this context is also poorly understood.

**Methods:**

To investigate the interplay between BCG, EVs, and bladder cancer, bladder cancer (BC) cell lines, dendritic cell (DC) and T cell co-culture systems, nanoparticle tracking analysis, molecular profiling, and Western blotting approaches were employed *in vitro*. Subcutaneous MB49 tumor models in mice were additionally used to assess the impact of exogenously administered BCG-induced EVs on DC activation, T cell proliferation, and antitumor efficacy *in vivo*.

**Results:**

We identified a novel mechanism of action involving BCG-induced EVs (EVs^BCG^). Following BCG internalization by bladder cancer (BC) cells, a robust immune cascade is triggered, accompanied by a surge in EV secretion and altered cargo composition. These EVs^BCG^ were found to be enriched with key immunomodulatory proteins, including MHC class I and II molecules as well as co-stimulatory molecules CD80 and CD86. Functionally, we found that EVs^BCG^ were capable of activating DCs, thereby enhancing T cell activity *in vitro*. Moreover, pre-treatment of mice with EVs^BCG^ significantly potentiated the therapeutic efficacy of BCG in a syngeneic murine bladder cancer model.

**Conclusion:**

These findings implicate EVs^BCG^ as critical intermediaries in the anti-tumor immune response and suggest their potential utility as mediators and/or predictive biomarkers for therapeutic efficacy. In summary, we propose a previously unrecognized mechanism of BCG immunotherapy mediated by EVs, establishing them as pivotal components of the immune signaling network driving effective treatment outcomes.

## 1 Introduction

Bladder cancer (BC) ranks as the fourth most common malignancy among males in the United States, and represents one of the most costly cancers to manage over a patient’s lifetime. The high economic burden, estimated at over $185,000 per patient (1), and $9.4 billion in annual national expenditures (2), is largely due to the necessity for long-term surveillance and repeated interventions for tumor recurrence and progression. Approximately 70% of newly diagnosed BC cases are non-muscle invasive (NMIBC), with recurrence rates exceeding 70% (3), and progression to muscle-invasive disease occurring in up to 30-50% of cases (4).

Since its introduction more than four decades ago, Bacillus Calmette-Guérin (BCG) has remained a cornerstone adjuvant therapy for high-grade NMIBC following transurethral resection of bladder tumor (TURBT). Originally developed in the early 20th century as a vaccine against tuberculosis (5, 6), BCG was later repurposed for oncology after early studies demonstrated its anti-tumor effects, including the suppression of metastatic melanoma (7). The first successful application of intravesical BCG in BC treatment was reported in 1976 (8), and it was subsequently approved by the U.S. Food and Drug Administration in 1990 as the standard of care for high-grade NMIBC (9). Despite its clinical efficacy, BCG therapy remains largely unchanged, and many aspects of its immunological mechanisms remain unresolved (10–12). Briefly, BCG is preferentially taken up by higher-grade tumor cells and can trigger the induction of both robust innate and adaptive immune responses locally and systemically upon intravesical administration (13), leading to activation of pattern recognition receptors that trigger NF-κB and MAPK signaling, resulting in the production of pro-inflammatory cytokines and chemokines that recruit and activate neutrophils, macrophages, and NK cells. The robust innate immune response that results can facilitate tumor clearance and elicit adaptive immunity to sustain therapeutic efficacy (11, 12). In addition to indirect immune-mediated cytotoxicity, BCG may exert direct effects on tumor cells, including induction of apoptosis, cell cycle arrest, and enhanced antigen presentation (14, 15). Recent work also suggests that BCG may colonize the bone marrow following intravesicle administration, reprogramming host immunity to favor tumor clearance (16). A deeper understanding of these mechanisms and their associations with treatment outcomes is crucial for optimizing treatment protocols and identifying biomarkers to predict therapeutic response and guide patient stratification.

Extracellular vesicles (EVs) are increasingly recognized as complex and dynamic mediators of intercellular communication and immune activity (17, 18). EVs are nanoscale, lipid bilayer-bound vesicles (30–250 nm in diameter) that carry diverse bioactive cargoes including proteins, lipids, nucleic acids, and carbohydrates (19). Initially considered cellular waste byproducts, EVs are now recognized as critical vehicles in both physiological and pathological processes (20–23), including immune surveillance, inflammation, and tumor progression (24). The immunological relevance of EVs was first recognized in 1996, when B cell-derived EVs were shown to present peptide-loaded MHC class II molecules (25), thereby functioning as antigen-presenting entities. Further studies revealed that EVs can be internalized by recipient cells and elicit downstream immune responses (17, 18). In the context of BC, tumor-derived EVs (TEVs) have primarily been studied for their tumor-promoting properties (26, 27), triggering immunosuppression and cancer progression (27). However, our recent findings demonstrated that BC-derived EVs can elicit anti-tumor immune responses and serve as effective cancer vaccines (28). Building on this, we hypothesize that EVs released by BC cells in response to BCG exposure, termed EVs^BCG^, may play a central role in mediating the immunostimulatory effects of BCG. While BCG-derived EVs have been explored as an alternative therapeutic option in place of mycobacterial administration (29), and limited efforts have been made to characterize BCG-induced changes in the microRNA profiles in macrophage-derived EVs (30), the functional roles of BCG-infected BC cell-derived EVs have yet to be characterized in detail.

In this study, we investigated the immunological functions of EVs^BCG^ and their contribution to BCG-induced anti-tumor immunity. We found that BCG stimulation significantly alters the composition of EVs secreted by BC cells, enriching them with immune-relevant cargo, including MHC molecules and co-stimulatory ligands. These EVs^BCG^ were capable of activating antigen-presenting cells and promoting CD8^+^ T cell proliferation *in vitro*. Using an *in vivo* syngeneic model, we further determined that EVs^BCG^ administration enhances BCG treatment efficacy. These findings collectively identify EVs^BCG^ as novel mediators of immunotherapy and lay the foundation for EV-based biomarkers and adjunctive strategies in bladder cancer treatment.

## 2 Materials and methods

### 2.1 Cell culture

The murine MB49 BC cell line was obtained from the American Type Culture Collection (ATCC) and cultured in Dulbecco’s Modified Eagle Medium (DMEM) supplemented with 10% fetal bovine serum (FBS, Thermo Fisher Scientific) and 1% penicillin/streptomycin (P/S, Gibco^®^, REF 15140-122). Cells were maintained at 37 °C in a humidified 5% CO_2_ incubator. The UMUC-3 human BC cell line and its BCG-resistant derivative UMUC-3-R were kindly provided by Dr. Hiroshi Miyamoto (University of Rochester Medical Center, Rochester, NY). These were cultured under identical conditions as MB49 cells. Additional human BC cell lines (T24 and RT4) were also acquired from ATCC and maintained in RPMI-1640 supplemented with 10% FBS and 1% P/S, in a 37 °C humidified 5% CO_2_ incubator.

### 2.2 Bacterial culture and treatment

TICE^®^ BCG was resuspended in 3 mL of DPBS, aliquoted into 20–100 µL volumes, and stored at -20 °C. The post-resuspension concentration was approximated at 1×10^6^ CFU/mL, based on manufacturer specifications. For in-house propagation, 20 µL (≈20 CFU) of BCG was inoculated into 10 mL Middlebrook 7H9 broth (Sigma-Aldrich, M0178) supplemented with Middlebrook OADC enrichment (BD, REF. 212351). Cultures were incubated in a shaker at 37 °C, 150 rpm for 21 days to reduce clumping. After incubation, cultures were sonicated for 15 min in a water bath with brief vortexing every 2–3 min. Samples were then allowed to settle for 10 min, and the upper fraction was transferred to 250 mL of fresh media for further expansion under identical conditions. Optical density at 600 nm (OD600) was measured every 2–3 days. At OD600 values ranging from 0.4 to 0.8 (in 0.1 increments), 1 mL samples were serially diluted and plated on Middlebrook 7H11 agar (Sigma-Aldrich, M0428) with OADC. Resulting colony counts were used to construct a standard growth curve correlating OD600 with CFU/mL. For experimental use, BCG cultures harvested at OD600 = 0.6 (~1×10^6^ CFU/mL) were centrifuged at 2400 rpm for 10 min, resuspended in DPBS to 1×10^6^ CFU/µL, aliquoted, and stored at -20 °C.

BC cell treatments were performed using 1×10^6^ CFU/mL BCG in EV-depleted FBS-containing medium without P/S. Treatment durations ranged from 4 to 72 h. For repeated weekly BCG treatments, MB49 and T24 cells were plated at ~20% confluency and treated for 72 hours with BCG, washed three times with PBS, and returned to culture for an additional 96 hours. This week-long treatment process was then repeated five additional times for a total 6-week treatment period. At the end of each week, cells were collected for EV and protein analysis, with 10% of the remaining cells being retained for continued culture and treatment. Cells were routinely monitored for growth, with no visible differences in growth or viability rates between the control and BCG-treated groups.

### 2.3 Quantification of BCG internalization by PCR

BC cells (1 × 10^5^) were seeded in 24-well plates one day before treatment. Cells were then treated with BCG (1×10^6^ CFU/mL) for 4 h at 37 °C, washed thoroughly with PBS to remove extracellular BCG, and harvested for DNA extraction using the PureLink Genomic DNA Mini Kit (Invitrogen). PCR amplification was carried out using OneTaq DNA Polymerase (New England BioLabs) on a T100 Thermal Cycler (Bio-Rad) with the following cycling conditions: 95 °C for 3 min; 35 cycles of 95 °C for 15 sec, 55 °C for 30 sec, 68 °C for 30 sec. Primers for BCG-specific genes and GAPDH were used (sequences available upon request). PCR products were separated by electrophoresis on 3% agarose gels in TBE buffer containing 0.002% ethidium bromide, and visualized using a ChemiDOC MP imaging system.

### 2.4 qPCR

Human and murine BC cells were treated with 1×10^6^ CFU/mL BCG for 24 h at ~75% confluency. Total RNA was extracted using acid guanidinium thiocyanate-phenol-chloroform and quantified with a NanoDrop spectrophotometer (Thermo Scientific). cDNA synthesis was performed using 1 µg of RNA and the iScript cDNA synthesis kit (Bio-Rad) in a 20 µL reaction volume. qPCR was performed in triplicate using iQ SYBR Green Supermix (Bio-Rad). Target genes for human cells included those encoding IL-1β, IL-6, IL-8, CD80, CD86, H2-L, H2-Aa, and H2-Aa1; housekeeping genes included those encoding GAPDH, GOT1, and β-actin. For mouse MB49 cells, targets genes included those encoding IL-1β, IL-6, IL-8, CD80, CD86, HLA-A, HLA-B, HLA-DMA, and HLA-DMB; internal controls included genes encoding UBC and EEF1D. Gene expression was normalized to the internal controls and presented as relative expression levels.

### 2.5 EV isolation and nanoparticle tracking analysis

For EV collection, MB49 and T24 cells were cultured in media containing EV-depleted FBS, as previously described. Supernatants were centrifuged at 400 g for 10 min, followed by 10,000 g for 30 min to remove cells and debris, then stored at -80 °C. EVs were isolated by ultracentrifugation at 100,000 g for 2 h at 4 °C, washed with DPBS, and ultracentrifuged again under the same conditions. Final pellets were resuspended in DPBS and further cleared by centrifugation at 10,000 g for 5 min to remove aggregates. Protein concentrations of EV samples were measured using the Micro BCA Assay (Thermo Fisher Scientific, Catalog No. 23235) and stored at -80 °C. Particle size and concentration were assessed by NanoSight NS300 (Malvern Instruments) following 1:1000 dilution in DPBS. Each sample was recorded in five 30-second videos using a syringe pump with a flow speed set to 25, and the mode particle size and particles per cell per hour were calculated. Camera settings and particle detection thresholds were selected as appropriate for each batch of samples, and were remained constant for all samples in a given experiment.

### 2.6 Western blotting

Protein concentrations of whole cell lysates (WCL) and EV samples were determined using the Micro BCA Protein Assay Kit (Thermo Fisher Scientific, Catalog No. 23235). Samples (10–15 µg) were resolved on 10–12% SDS-PAGE gels, transferred onto polyvinylidene fluoride (PVDF) membranes, and probed with the following primary antibodies: B7-1 (1:300, H-208, sc-9091, rabbit polyclonal IgG, Santa Cruz Biotechnology), B7-2 (1:300, BU63, sc-19617, mouse monoclonal IgG_1_, Santa Cruz Biotechnology), MHC Class I (1:700, H-300, sc25619, rabbit polyclonal IgG, Santa Cruz Biotechnology), MHC Class II I-A/I-E (1:200, M5/114.15.2, 14-5321-81, monoclonal IgG2b, eBioscience™), GAPDH (1:5000, 6C5, sc-32233, mouse monoclonal IgG_1_, Santa Cruz Biotechnology) and the EV marker PDCD6IP-Alix (1:300, 12422-1-AP, polyclonal IgG, PROTEINTECH^®^ GROUP). Secondary antibodies included donkey anti-rabbit IgG-HRP (1:5000, sc-2313) and goat anti-mouse IgG-HRP (1:4000, sc-2005), both from Santa Cruz Biotechnology. Signals were detected using SuperSignal™ West Femto Maximum Sensitivity Substrate (Thermo Scientific, REF. 34096) and visualized by chemiluminescence.

### 2.7 Murine DC differentiation, treatment, and co-culture

Bone marrow (BM) cells were differentiated *in vitro* into DCs as previously described (28). Briefly, BM was harvested from the femurs of individual 8- to 12-week-old female C57BL/6 mice. A total of 2 × 10^6^ BM cells were cultured in 10 mL RPMI 1640 medium supplemented with 10% fetal bovine serum (Thermo Fisher Scientific), 1% penicillin/streptomycin (Gibco^®^, REF 15140-122), 50 µM 2-mercaptoethanol (Aldrich^®^ Chemistry, REF M6250), 20 ng/mL recombinant murine GM-CSF (R&D Systems, Cat. 415-ML), and 5 ng/mL recombinant murine IL-4 (R&D Systems, Cat. 404-ML). Cultures were maintained at 37 °C with 5% CO_2_ in a humidified incubator. On days 3 and 5, an additional 10 mL of fresh supplemented medium was added. By day 7, loosely adherent cells were harvested, counted, and reseeded at the required density. The following day, BM-derived DCs were treated with either PBS (negative control), 1 × 10^6^ CFU/mL BCG (positive control), 100 µg/mL MB49-derived extracellular vesicles (EVs), or 100 µg/mL MB49 EVs obtained following three rounds of BCG priming (EVs^3rd^ BCG). Cells were incubated for 24 h. Following treatment, cells were collected, washed, and stained for flow cytometric analysis using the following antibodies: anti-mouse CD45 (PE/Cyanine7, clone 30_F11, BD Biosciences, Cat. 561868), CD11c (Brilliant Violet 605™, clone N418, BioLegend, Cat. 117333), MHC class II I-A/I-E (Alexa Fluor^®^ 700, clone M5/114.15.2, BioLegend, Cat. 107621), and CD86 (APC/Cyanine7, clone GL-1, BioLegend, Cat. 105029). DCs were identified as CD45^+^ CD11c^+^, and activation status was assessed based on surface expression levels of MHC class II and CD86. For co-culture assays, pre-treated DCs were co-cultured at a 1:10 ratio with CD8^+^ T cells isolated from the spleens of two 8–12-week-old female C57BL/6 mice previously primed subcutaneously with 20 µg MB49 EVs and 1 × 10^6^ CFU BCG (administered twice at days -14 and -21 before harvest). CD8^+^ T cells were isolated using the EasySep™ Mouse CD8^+^ T Cell Isolation Kit (Stemcell Technologies, Cat. 19853A), per the manufacturer’s protocol. Purified T cells were labeled with CFSE (CellTrace™ CFSE Cell Proliferation Kit, Invitrogen, REF C34554), according to the manufacturer’s instructions, and co-cultured with BMDCs for 5 days. Post co-culture, cells were harvested, washed, and stained for flow cytometric analysis using anti-mouse CD45 (PE/Cyanine7, BD Biosciences), CD11c (Brilliant Violet 605™, BioLegend), and CD8 (PE, clone 53-6.7, Invitrogen, REF 12-0081-82). CD8^+^ T cells were gated as CD45^+^ CD8^+^ CFSE^+^ and their proliferation was assessed via CFSE dilution and visualized as histograms.

### 2.8 Human DC differentiation, treatment, and co-culture

THP-1 monocytes and Jurkat T cells were obtained from ATCC (Cat. TIB-202 and TIB-152, respectively) and cultured in RPMI 1640 supplemented with 10% FBS (Thermo Fisher Scientific) and 1% penicillin/streptomycin (Gibco^®^, REF 15140-122) at 37 °C in a humidified atmosphere containing 5% CO_2_. THP-1 cells were differentiated into DC-like cells by culturing them in medium supplemented with 50 µM 2-mercaptoethanol, 100 ng/mL recombinant human GM-CSF (R&D Systems, Cat. 215-GM), and 100 ng/mL recombinant human IL-4 (R&D Systems, Cat. 204-IL). Fresh media was added on days 3 and 5. On day 7, non-adherent cells were collected, counted, and reseeded. On day 8, cells were treated with PBS, 1 × 10^6^ CFU/mL BCG, 100 µg/mL T24 EVs, or 100 µg/mL T24 EVs^3rd^ BCG and incubated for 24 h. Cells were then collected, washed, and stained for flow cytometry using the following antibodies: anti-human CD11c (Brilliant Violet 650™, clone Bu15, BioLegend, Cat. 337237), MHC class II HLA-DR (PE, BD Pharmingen™, Cat. 555812), and CD86 (PE/Cyanine7, clone IT2.2, BioLegend, Cat. 305422). DCs were gated as CD11c^+^, and activation was evaluated based on MHC class II and CD86 expression. For co-culture, pre-treated THP-1-derived DCs were incubated with CFSE-labeled Jurkat T cells at a 1:10 ratio. Jurkat cells were labeled with CFSE (CellTrace™ CFSE Cell Proliferation Kit, Invitrogen, REF C34554) per manufacturer’s instructions and co-cultured with DCs for 5 days. After co-culture, cells were harvested, stained with anti-human CD11c (BioLegend), and analyzed by flow cytometry. Jurkat T cells were identified as CD11c^−^ CFSE^+^, and their proliferation was quantified based on CFSE dilution and displayed as histograms.

### 2.9 *In vivo* tumor inoculation, EV priming, and BCG immunotherapy

For *in vivo* tumor studies, 8- to 12-week-old female C57BL/6J mice were subcutaneously inoculated in the back with 2 × 10^5^ MB49 tumor cells suspended in 100 µL PBS using 23G needles. Mice were randomly assigned into three groups (n = 5/group) and, on days 7 and 14 post-tumor implantation, received subcutaneous priming (50 µL into the flank using 26G needles) with either: DPBS (control), 20 µg MB49 EVs, or 20 µg MB49 EVs^3rd^ BCG. Subsequently, on days 10, 17, and 24, mice from each priming group were further randomized to receive intratumoral injections (20 µL) of either 1 × 10^6^ CFU/mL BCG or PBS control using 26G needles. Tumor growth was monitored over time, and mice were euthanized when tumors reached ethical endpoint size limits.

## 3 Results

### 3.1 BCG uptake by BC cells induces immune priming and EV release

TICE^®^ BCG has been the sole strain available to treat NMI BC patients in the United States and much of Europe since 2017 (31, 32), precipitating a shortage in TICE^®^ BCG stocks that has limited their research availability (33), in addition to reducing BCG utilization rates in patients (34). To enable the present study, we cultured BCG in-house (**Supplementary Figure 1A**). When used to stimulate human T24 and murine MB49 BC cells *in vitro*, our in-house BCG and TICE^®^ BCG elicited comparable mRNA-level induction of key cytokines, including *IL1B, IL6*, and *IL8 (*35–38) (**Supplementary Figures 1B, C**).

**Figure 1:**
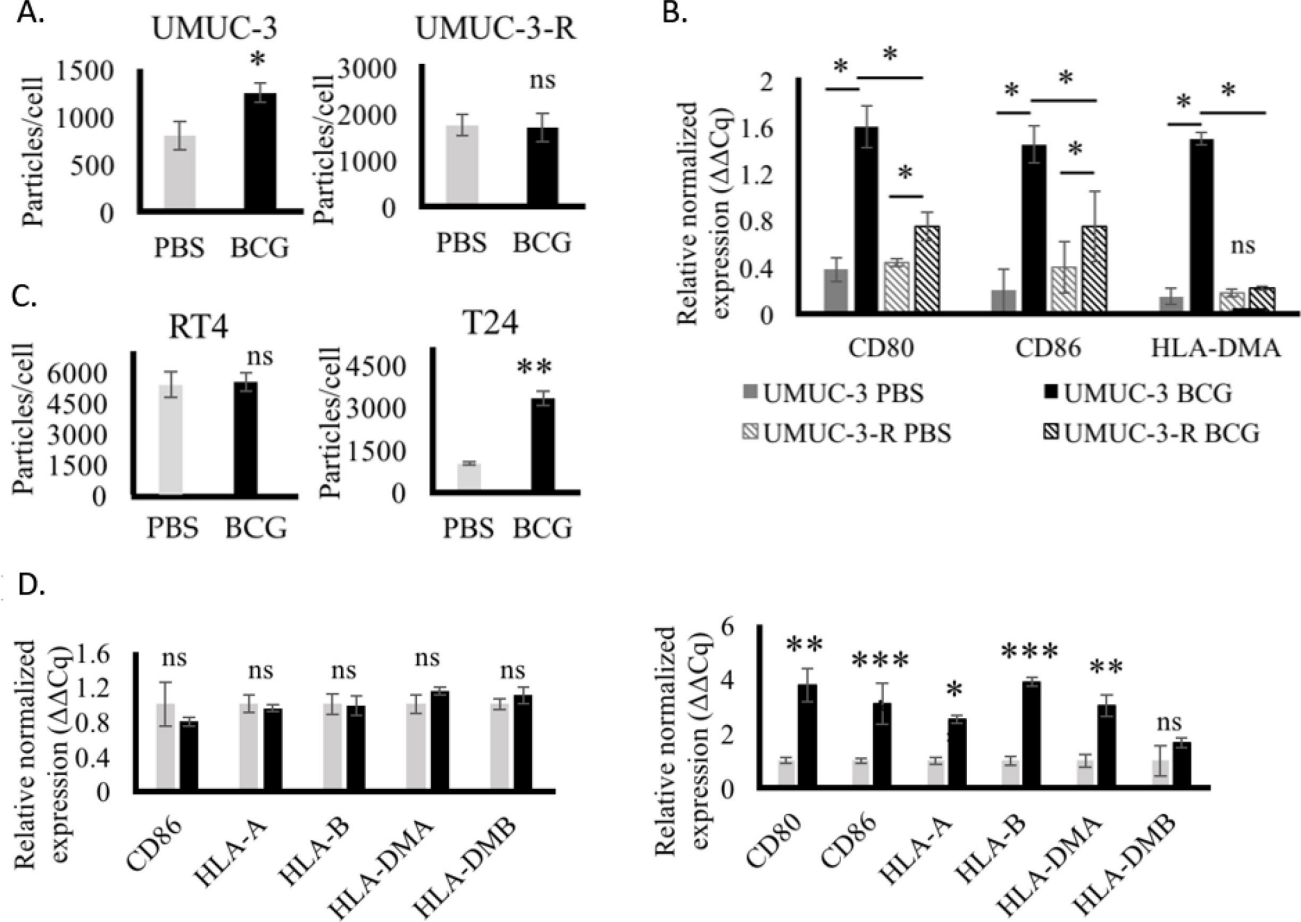
BCG internalization increases EV release by BC cells. **(A)** EV release from UMUC-3 and UMUC-3-R cells after BCG treatment for 24 h as measured by NTA. **(B)** qPCR analysis of the expression of the indicated immunomodulatory genes in human UMUC-3 and UMUC-3-R BC cells after BCG treatment for 24 h. **(C)** EV release from RT4 and T24 cells after treatment for 24 h with BCG, as measured by NTA. **(D)** qPCR analysis of the expression of the indicated immunomodulatory genes in RT4 and T24 cells after BCG treatment for 24 h. Data are means ± standard deviation. *P < 0.05, **P < 0.01, ***P < 0.001; ns, not significant. Student’s t-tests or two-way ANOVAs with Tukey’s multiple comparisons test. All experiments were independently repeated three times, with n = 3–5 replicates per group.

The uptake of BCG is reportedly essential for its immunogenic effects within BC cells (39). Nanoparticle tracking analysis (NTA) of supernatants from BCG-treated BC cells revealed that BCG inoculation elicited enhanced EV release from the human UMUC-3 BC cell line (**Figure 1A**), whereas no such induction was apparent for the BCG-resistant UMUC-3-R subline (40), in line with prior evidence that various stress-related stimuli can provoke EV secretion (41–43). The BCG-driven upregulation of key immunostimulatory genes was similarly dependent on internalization in UMUC-3 cells (**Figure 1B**). To extend these results to additional cellular models, two human cell lines that reportedly exhibit low (RT4) or high (T24) levels of BCG uptake (44) were treated with BCG. In line with results from UMUC-3 cells, BCG enhanced EV release from T24 but not RT4 cells (**Figure 1C**), in addition to upregulating key MHC and co-stimulatory genes in T24 but not RT4 cells (**Figure 1D**). Together, these data reaffirm the uptake-dependent initiation of an immunostimulatory response in BC cells that coincides with enhanced EV biogenesis.

### 3.2 BCG induces the secretion of immunostimulatory EVs from BC cells

**Figure 2:**
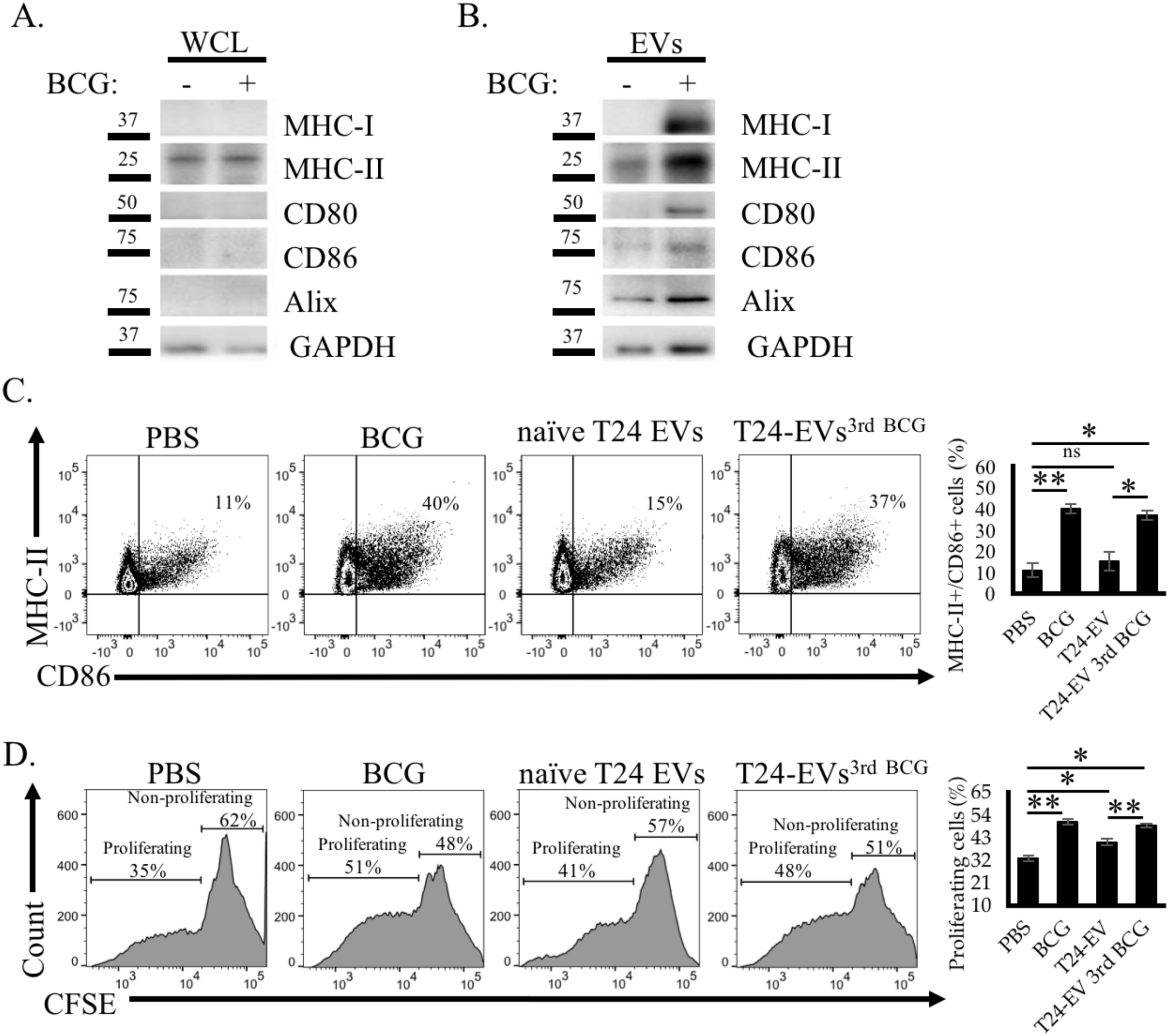
Immunostimulatory T24 EVs^BCG^ activate DC and T cell responses *in vitro*. **(A, B)** Western blotting analysis of the expression of different immunomodulatory proteins in T24 whole cell lysate (WCL) and EV samples collected before and after BCG treatment for 72 h. **(C)** Flow cytometry analyses of the activation of THP-1 DCs after T24 EVs^3rd BCG^ treatment for 24 h, with corresponding quantification of MHC-II^+^/CD86^+^ cells. **(D)** The proliferation of CFSE-stained human Jurkat T cells co-cultured with various EV pre-treated THP-1 DCs for five days was quantified via flow cytometry, with corresponding quantification of proliferating cell frequencies. Data are means ± standard deviation. *P < 0.05, **P < 0.01, ns, not significant, two-way ANOVAs with Tukey’s multiple comparisons test. All experiments were independently repeated three times, with n = 3–5 replicates per group.

EVs have been reported to modulate immune cell activity in part through MHC-mediated antigen presentation and costimulatory protein (CD80, CD86) expression (45, 46). Strikingly, Western blotting revealed that EVs derived from BCG-treated T24 cells (T24 EVs^BCG^) harbor higher levels of MHC-I, MHC-II, CD80, and CD86 than those from unstimulated cells, whereas no clear BCG-related differences in the levels of these proteins were apparent in whole T24 cell lysates (**Figures 2A, B**). To assess the immunostimulatory potential of these T24 EVs^BCG^, monocytic THP-1 cells were differentiated into DC-like cells (THP-1-DCs) (47), followed by treatment with T24 EVs^BCG^, naïve T24 EVs, or direct BCG stimulation. Flow cytometry revealed that treating THP-1 DCs with T24 EVs^BCG^ or BCG, but not naïve T24 EVs, significantly increased the frequency of activated (MHC-II^+^/CD86^+^) cells (**Figure 2C**). Consistent with their enhanced activation, these EV^BCG^-activated THP-1-DCs were better able to activate Jurkat T cell proliferation *in vitro* in a carboxyfluorescein succinimidyl ester (CFSE) dilution assay (**Figure 2D**). These results suggest that BCG treatment enhances EV release from BC cells and that these immunogenic EVs are capable of directly activating DCs and thereby indirectly activating T cells, suggesting a unique role for these BCG-induced EVs as modulators of the immune responses induced in BC patients treated with BCG.

### 3.3 BCG induces the release of EVs that contain immunostimulatory molecules and enhance recipient CD8^+^ T cell responses

**Figure 3:**
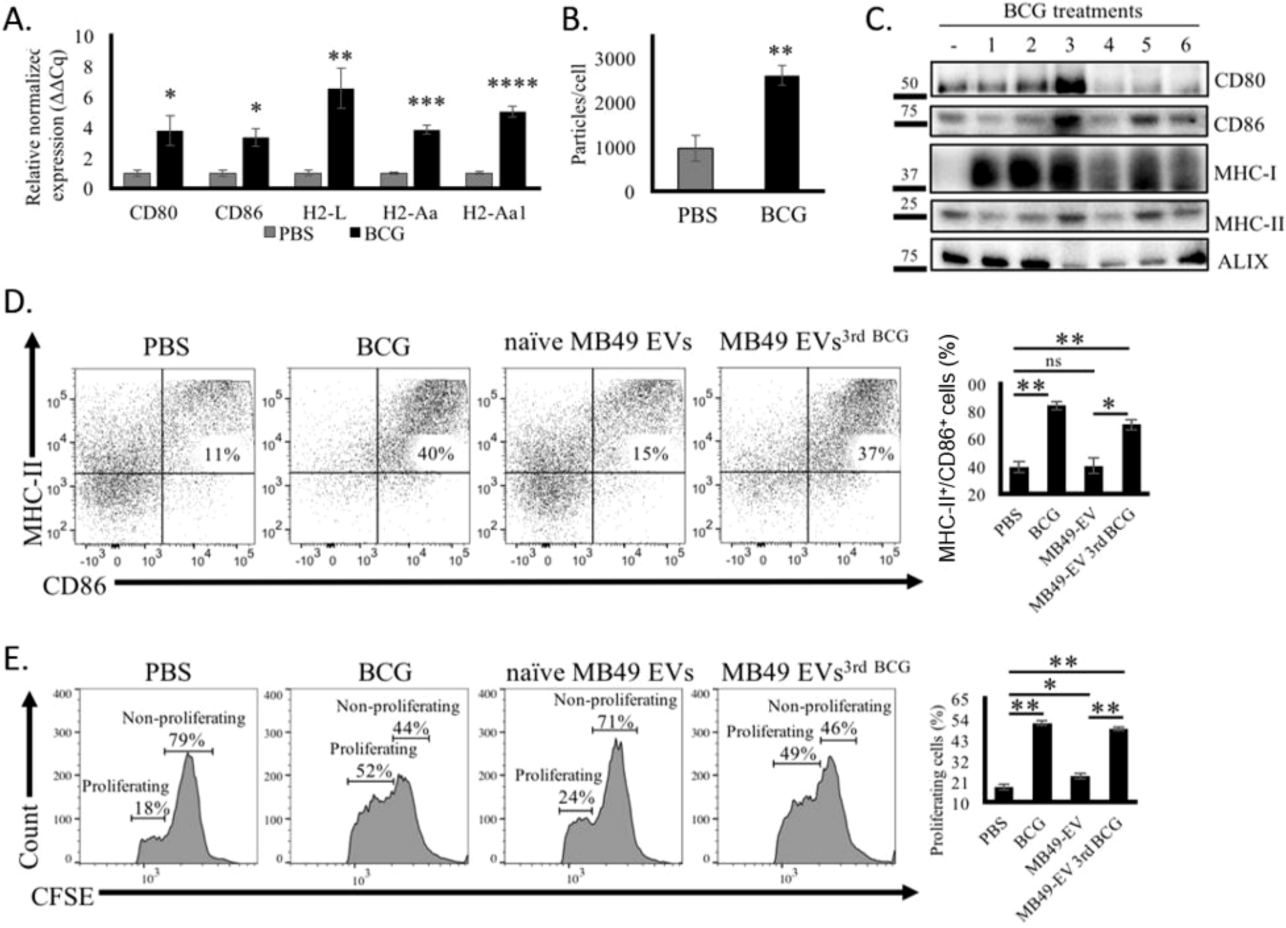
BCG treatment enhances the release of immunostimulatory EVs from murine BM cells. **(A)** qPCR analysis of the expression of different immunomodulatory genes in murine MB49 BC cells following BCG treatment for 24 h. **(B)** EV release from MB49 cells after treatment with BCG for 24 h, as measured by NTA. **(C)** Western blotting analysis of different immune-related proteins and EV markers in EV samples collected from MB49 cells at 72 h after each of 6 weekly BCG treatments. **(D)** Flow cytometry analyses of the activation of BMDCs after MB49 EVs^3rd BCG^ treatment for 24 h, with corresponding quantification of MHC-II^+^/CD86^+^ cells. **(E)** Flow cytometry analysis of the proliferation of CFSE-stained murine CD8^+^ T cells isolated from BCG and MB49 EV-primed mice after co-culture with pre-treated BMDCs for five days, with corresponding quantification of the frequency of proliferating CD8^+^ T cells. Data are means ± standard deviation. *P < 0.05, **P < 0.01, ***P < 0.001, ****P < 0.0001, ns, not significant, Student’s t-tests or two-way ANOVAs with Tukey’s multiple comparisons test. All experiments were independently repeated three times, with n = 3–5 replicates per group.

To extend these analyses, the syngeneic MB49 murine BC cell line was chosen as a target for further study. *In vitro*, BCG treatment induced immunostimulatory gene upregulation and enhanced EV release in these MB49 cells (**Figures 3A, B**), consistent with the results observed in human cells above. When MB49 cells were treated once per week with BCG for 6 weeks (See Section 2.2 for details), peak CD80, CD86, MHC-I, and MHC-II protein levels were observed in EVs harvested after the third round of treatmentt (**Figure 3C**).

In line with the results observed using human cells, MB49 EVs^BCG^ significantly increased the frequency of activated (MHC-II^+^/CD86^+^) murine bone marrow-derived DCs (BMDCs) relative to PBS treatment (11% vs. 37%), similar to the activation observed among BMDCs directly treated with BCG (40%), whereas naïve EVs failed to activate these cells (**Figure 3D**). Finally, to test the ability of these EV^BCG^-treated DCs to activate T cells, we isolated CD8^+^ T cells from the BCG and EVs^BCG^-primed animals and combined them with these BMDCs. EV^BCG^-primed BMDCs induced more robust CD8^+^ T cell proliferation in a CFSE dilution assay as compared to PBS- or naïve EV-primed DCs (**Figure 3E**). These results strongly suggest that the immunostimulatory EVs^BCG^ can enhance DC-mediated T cell activation, supporting the potentially crucial role that EVs may play as mediators of BCG immunotherapy.

### 3.4 MB49 EV^BCG^ priming enhances the antitumor efficacy of BCG in mice

**Figure 4:**
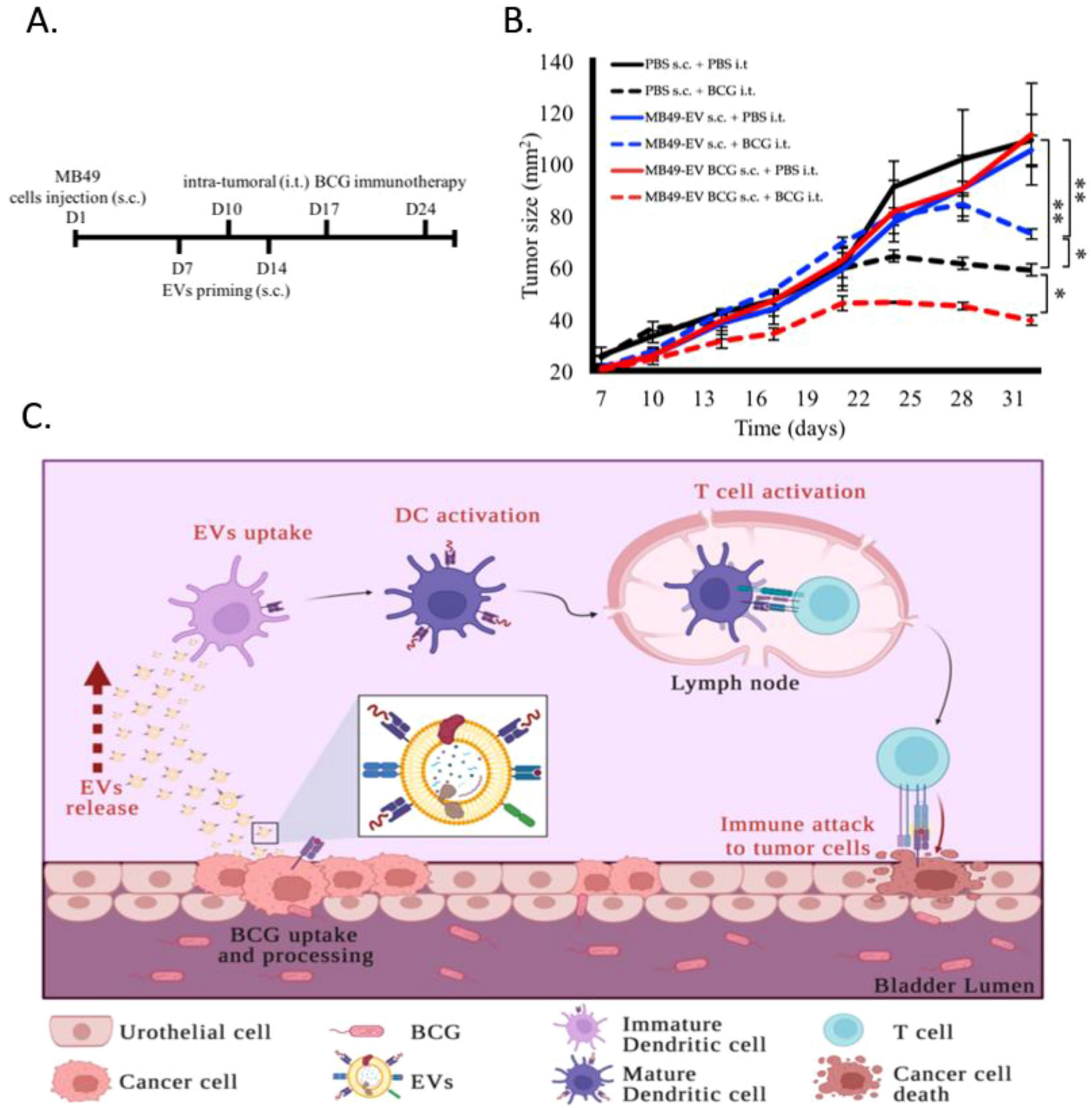
BCG-induced EVs enhance the antitumor efficacy of BCG in mice. **(A, B)** Timeline for the *in vivo* EV priming and BCG treatment experiment in MB49 tumor-bearing mice (n=5/group) **(A)** and corresponding analyses of tumor growth over time **(B)**. **(C)** Proposed role for EVs as mediators of the efficacy of BCG immunotherapy. Following primary tumor removal and the intravesical administration of BCG, these mycobacteria are internalized by remaining BC cells through the α5β1 integrin-bacterial fibronectin interaction that drives macropinocytosis in BC cells. Once BCG is internalized, BC cells increase the release of immunoactive EVs into the bladder. Immuno-active EVs are later internalized by APCs, including DCs, resulting in their activation. Activated DCs interact with T cells in lymph nodes to trigger their activation through immunological synapse formation. These activated T cells can then identify BC cells and perform their effector functions, resulting in tumor cell death and clearance (Figure created with BioRender.com). Data are means ± standard deviation. *P < 0.05, **P < 0.01, Student’s t-tests or two-way ANOVAs with Tukey’s multiple comparisons test. All experiments were independently repeated three times.

To gain further insight into the functional roles of BC EVs^BCG^ in a more physiologically relevant context as regulators of the efficacy of BCG immunotherapy outcomes, C57BL/6 mice bearing subcutaneous MB49 tumors were primed with PBS, naïve EVs, or EVs^BCG^ on days 7 and 14 following tumor implantation. Then, the MB49 tumor-bearing mice were randomly assigned into two groups that received weekly intratumoral (i.t.) BCG or PBS treatment (**Figure 4A**), monitoring tumor growth over time. We found that BCG treatment alone significantly suppressed tumor growth relative to PBS control in mice primed with PBS (**Figure 4B**). While MB49 EV^BCG^ treatment alone failed to suppress tumor growth, the combined administration of BCG and EVs^BCG^ resulted in significant tumor growth suppression relative to BCG alone, or to the combination of BCG + naïve EV treatment. These data emphasize the immunogenic nature of these BCG-induced tumor cell-derived EVs, as they can enhance the ability of BCG immunotherapy to inhibit tumor growth.

## 4 Discussion

Effective and sustained communication between tumor cells and the immune system is fundamental to the therapeutic success of BCG immunotherapy. Our analyses revealed the key mechanistic insight that BC cells that internalize BCG exhibit enhanced secretion of EVs enriched for key immunomodulatory molecules. These EVs, which we refer to as EVs^BCG^, carry MHC class I and II molecules, co-stimulatory proteins (CD80 and CD86), and potentially other immunologically active factors. Importantly, these vesicles are not merely by-products of BCG exposure, instead serving as biologically active agents capable of modulating the anti-tumor immune response (**Figure 4C**). This discovery positions EVs as active mediators in BCG-induced immunological reprogramming, thereby identifying a previously underappreciated pathway contributing to treatment efficacy.

It has been found that BCG initiates anti-tumor immunity by adhering to and being internalized by urothelial cells through interactions with integrin α5β1 and its bacterial fibronectin ligand (39, 48). This internalization stimulates local immune activation and inflammatory signaling (49). Consistent with this model, we found that only BC cell lines capable of robust BCG internalization, including T24 and parental UMUC-3 cells, exhibited significantly increased EV release following BCG exposure. In contrast, RT4 cells, which lack α5 integrin expression (50), and UMUC3-R cells, engineered for BCG resistance (40), failed to show such a response. These findings establish a functional link between BCG internalization capacity and the secretion of immunogenic EVs. Functionally, we further demonstrated that EVs^BCG^ could activate DCs and promote CD8^+^ T cell proliferation *in vitro*, highlighting their potential role in priming anti-tumor immunity. Crucially, these effects translated to *in vivo* activity, enhancing the antitumor efficacy of BCG in tumor-bearing mice and thereby underscoring the immunomodulatory potential of these BCG-induced EVs in tumor-bearing animals.

BCG remains an important form of therapy for patients with high-grade NMIBC, yet a large proportion of patients ultimately fail to respond (52, 53). Current alternative immunotherapeutic options, including checkpoint inhibitors, have shown limited efficacy in this context (51). Therefore, it is critical to deepen our understanding of the mechanism of action of BCG and to identify biomarkers predictive of patient responses to inform the development of novel immunotherapeutic interventions in BC. Our study contributes to this effort by revealing a new mechanism through which BCG exerts EV-mediated immunomodulatory effects, providing evidence for the functional role of these vesicles in immune activation. The specific cargos within these EVs that mediate their observed therapeutic efficacy remain uncertain, though we speculate that the MHC-I/II and CD80/86 molecules we were able to detect within EVs^BCG^ may have contributed to these effects, given the importance of MHC molecules for T cell activation and the role of CD80/86 as essential costimulatory molecules. Prior research suggests that BCG can modulate the microRNA profiles present within macrophage-derived EVs upon infection (30), raising the possibility of microRNA-related regulatory activity in our experimental system. Other factors that may be present in EVs^BCG^ include damage-associated molecular patterns with the potential to trigger innate immunity and inflammation (52), heat-shock proteins (53), and various cytokines/chemokines (27). Through further efforts to characterize the relationships between specific cargo composition and patient outcome are needed, EVs^BCG^-associated signatures could serve as dual biomarkers and therapeutic adjuncts, informing both patient stratification and combination treatment strategies.

There are certain limitations to this study that should be taken into consideration. For one, EV treatment strategies implemented in this study were based on the protein content within the utilized EVs, rather than on particle number. While this strategy is routinely employed in our laboratory, in future analyses we will incorporate both normalization approaches to characterize EV-related effects on both a per-EV and per-protein basis. In addition, our results using Jurkat cells and THP-1-DCs, while informative and consistent with the murine phenotypes observed in this study, warrant validation using primary human leukocyte systems to ensure their broader applicability. Similarly, our mouse model studies require further validation with an orthotopic model of BC in the context of intravesical BCG administration to fully confirm their relevance to human disease. More extensive characterization of the immunomodulatory effects of EVs^BCG^ treatment should also be conducted by analyzing DCs and other immune cell populations in the tumor, tumor draining lymph nodes, and spleen of treated mice.

In summary, our study demonstrates that BCG induces the secretion of functionally reprogrammed EVs from responsive BC cells, and that these EVs facilitate immune cell activation and anti-tumor signaling. Our work identifies EVs as both biomarkers and active agents in BCG immunotherapy, opening new avenues for improving therapeutic efficacy and personalizing treatment in NMIBC.

## Data Availability

The original contributions presented in the study are included in the article/**Supplementary Material**. Further inquiries can be directed to the corresponding author.
